# The Totalitarianization of Language in Dictatorial Systems, the Loss of Meaning, and the Confinement of Humanity to Instinctual Life within a Social Darwinist Framework: The Aztek Syndrome

**DOI:** 10.1192/j.eurpsy.2026.11299

**Published:** 2026-07-31

**Authors:** H. Yildiz

**Affiliations:** psychiatry, Turkish Psychiatric Association, Ankara, Türkiye

## Abstract

**Introduction:**

This article examines the consequences of language being subjected to hegemony and totalitarianism in dictatorial systems on human existence. The severing of language from meaning leads to the erasure of social memory and the narrowing of the individual’s intellectual horizons. Ultimately, humans are reduced to mere instincts, trapped in an existence struggle within a social Darwinist life. This situation is termed the ‘Aztec Syndrome’ in the study; that is, the individual being stripped of thought, meaning, and freedom, confined solely to instinctual existence.

**Objectives:**

In th In is study, it is argued that the totalitarianisation of language in dictatorial societies leads to the closure of discourse and the linguistic universe, causing a loss of meaning in individuals. To fill this void of meaning, individuals develop various psychological defence mechanisms. The failure to meet the spiritual needs of individuals and social life creates existential crises and condemning individual and social life to a social Darwinist level. The aim is to understand individuals’ behaviour within society by analysing the dynamics of these psychological defence mechanisms and eliminating the conditions that cause them.

**Methods:**

A qualitative method was used in this study.

**Results:**

Consequently, in totalitarian societies, the closure of language’s discursive and semantic expansion has, in a sense, hindered the movement of the social mind and led to a serious loss of meaning in social and individual life. A natural consequence of this is to herd individuals into a social Darwinist existence, to make them passive, to condemn them to a purely instinctual biological existence, This loss of meaning has caused existential and spiritual crises in individual and social life, and to compensate for this, various psychological defence mechanisms have come to the fore in the daily life of individuals and society. Without understanding these defence mechanisms, it is impossible to understand the individual and social behaviour of a totalitarian society.

**Conclusions:**

The Aztec Syndrome is not merely a metaphor, but also a warning: the subjugation of language under hegemony disconnects humans from meaning and thought; ultimately condemning the individual to a purely instinctual existence. This represents a loss of human existence at both the individual and societal levels.

**Disclosure of Interest:**

None Declared

